# Focal palmoplantar and gingival keratosis – A rare genodermatoses: Case report

**DOI:** 10.4317/jced.57528

**Published:** 2020-11-01

**Authors:** Roberto Gerber-Mora, Martín Jajam-Maturana, Alejandra Fernández-Moraga, Rene Martinez-Flores

**Affiliations:** 1 Unidad de Patologia y Medicina Bucal, Universidad Latina de Costa Rica; 2 Departamento de Patologia Oral, Facultad de Odontologia, Universidad Andres Bello, Chile

## Abstract

Focal palmoplantar and gingival keratosis syndrome is a rare dominant inherited disease with an early onset in life. Clinically, the condition is characterized by pressure related thickening of the epidermis of the palms and soles, usually accompanied by pain and different levels of skin involvement and thickness between patients. Recently, we observed a 38-year-old woman with multiple non-removable, painless white plaques of variable size and thickness on the attached gingiva and a white plaque widespread across the hard palate. By further questioning, the patient comments that she has thick yellowish focal plaques in both soles of her feet. Histopathological analysis revealed a hyperplastic and hyperorthokeratinized stratified squamous epithelium with basal hyperplasia. The spinous, granular and stratum corneum showed dispersed basophilic keratohyalin granules. At higher magnification, the keratinocytes contained paranuclear bodies, seen as round eosinophilic condensation that indented the nuclei. Based on these findings the final diagnosis was rendered as focal palmoplantar and gingival keratosis.

** Key words:**Genodermatoses, white plaque, dyskeratosis, gingiva.

## Introduction

Recognized as part of an heterogenous group of palmoplantar keratodermal disorders (PKD) (1,2), focal palmoplantar and gingival keratosis syndrome (FPGKS) is a rare dominant inherited disease first reported by Fred *et al* in 1964 (3), although Gorlin characterized and defined this syndrome later in 1976, and since then, only a few cases have been reported, including cases of consecutively affected generations within the same family(4).

The onset of this disease generally occurs during childhood and puberty, with a tendency to worsen through life (1,3). Clinically, the condition is characterized by pressure related thickening of the epidermis of the palms and soles, usually accompanied by pain (4) and different levels of skin involvement and thickness between patients, most likely due to the type of occupation, weight, stature, exercise, and/or type of footwear used by the patient (5). The oral lesions are white plaques confined to the attached gingiva, hard palate or areas of chronic friction in the oral mucosa (6).

Since FPGKS can be easily mistaken with other types of PKD that also express intraoral alterations such as Papillon-Lefèvre syndrome (PLS) (palmoplantar keratoderma with periodontitis), or more importantly, disorders that represent a threat to the life of the patient by developing malignant neoplasms, like Howel-Evans syndrome (HES) (Tylosis with esophageal cancer) (1,2,5), it is imperative to understand the clinicopathological differences that help differentiate FPGKS from other diseases, in order to make the right diagnosis. Herein, we report the case of a 38-year-old woman with focal palmoplantar and gingival keratosis syndrome.

## Case Report

A 38-year-old woman, smoker (2 cigarettes a day), was referred to the oral medicine service at the Andres Bello University, for having “white spots” in her mouth. Her medical history revealed that the patient suffers from insulin resistance, for which she takes metformin, otherwise, her medical history is unremarkable.

**Figure 1:**
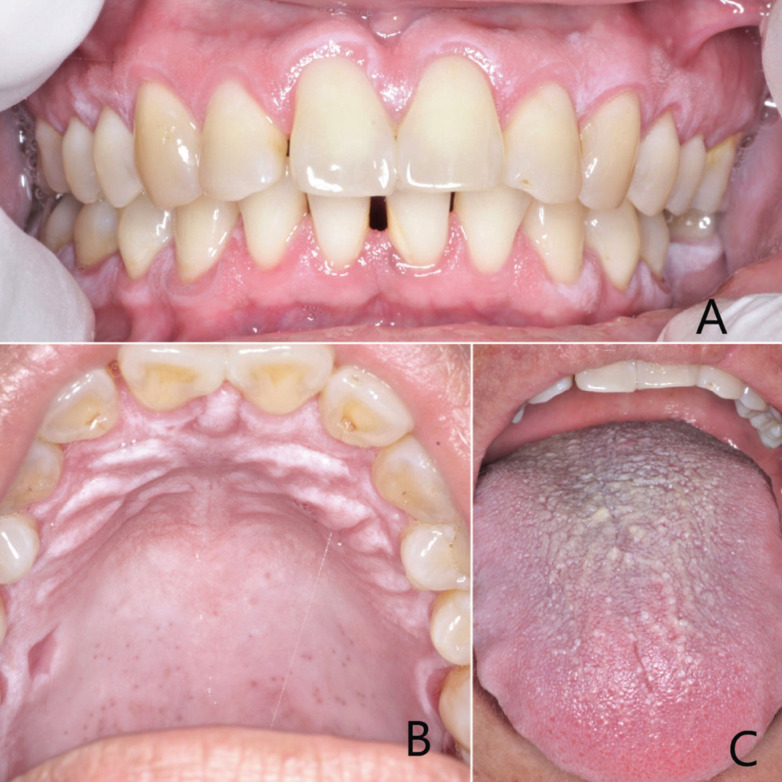
A) White plaques of variable size and thickness where observed in the attached gingiva. B) White plaque widespread across the hard palate sparing the free marginal gingival. C) Thickened and whiter than normal filiform papillae.

Upon intraoral examination, multiple non-removable, painless white plaques of variable size and thickness where observed in the attached gingiva, with no involvement of the free marginal gingivae and alveolar mucosa (Fig. 1A). Also, a similar white plaque widespread across the hard palate sparing the free marginal gingival was noticed (Fig. 1B). On the dorsal side of the tongue, the filiform papillae were elongated, thickened and whiter than normal, which gave the impression as if the tongue had a white coat covering it (Fig. 1C). No other alterations were observed.

**Figure 2:**
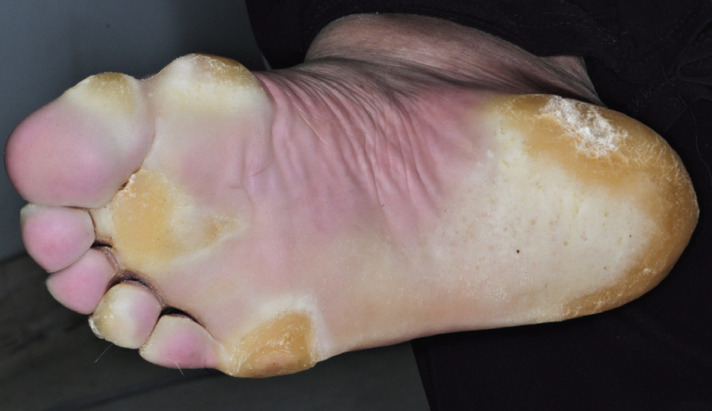
Thick yellowish focal plaques on the sole of the right foot.

By further questioning, the patient comments that she has never noticed anything out of the ordinary in her mouth, but she does have thick yellowish focal plaques in both of her soles (Fig. 2). No similar lesions were present in other parts of her body, including the palms of her hands. The patient also explains that her father and grandmother have similar lesions on palms and soles.

**Figure 3:**
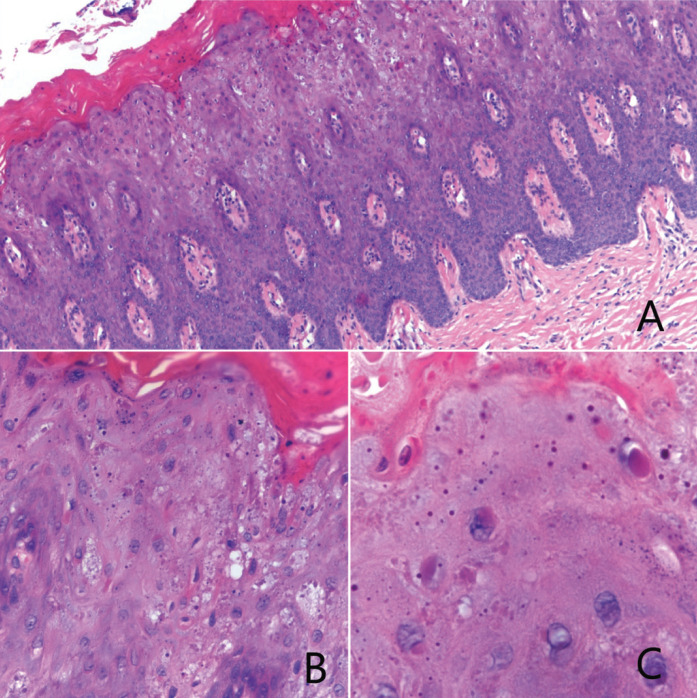
A) Hyperplastic and hyperorthokeratinized stratified squamous epithelium. B) Basophilic keratohyalin granules in granular and corneum stratum. C) Round eosinophilic condensation indenting nuclei.

Based on these findings and under the suspicion of a genodermatoses (Table 1), an incisional biopsy of the lesions in the hard palate and attached gingivae was performed. Histopathologically, both specimens revealed a hyperplastic and hyperorthokeratinized stratified squamous epithelium with basal hyperplasia containing multiple normal mitosis (Fig. 3A). The spinous, granular and corneum stratum showed dispersed basophilic keratohyalin granules (Fig. 3B). At higher magnification, the keratinocytes contained paranuclear bodies, seen as round eosinophilic condensation that indented the nuclei, giving it a coma or “Pac-man” shape (Fig. 3C). The connective tissue underneath was of normal appearance, demonstrating a dense collagenized chorion, containing multiple fibroblasts, hyperemic capillaries and the presence of chronic inflammatory cells.

**Table 1:**
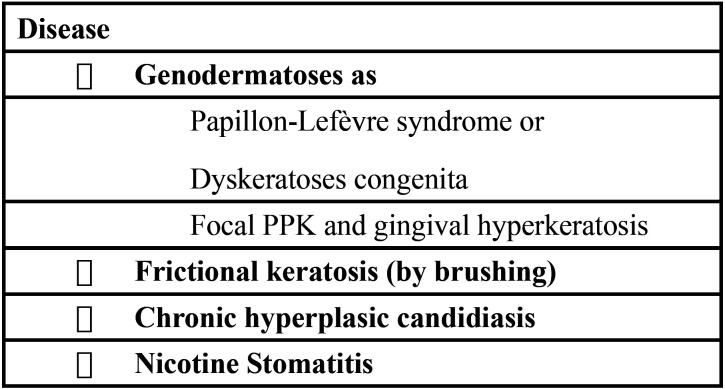
Differential diagnosis taken into consideration on the present case.

Upon these clinical and histopathological findings, the final diagnosis was rendered as focal palmoplantar and gingival keratosis disorder. The patient was referred to dermatology for further investigations.

## Discussion

The genodermatoses are a group of rare, inherited, single-gene skin disorders that are often associated with a variety of other medical abnormalities (2), resulting in the alteration of cellular mechanisms that lead to the expression of different clinical phenotypes on the skin and mucosae (7). Focal palmoplantar and gingival keratosis is an autosomal dominant disorder (OMIM *148730) were patients develop painful keratosis in palms and soles prompted by pressure and friction, along with marked hyperkeratosis of the attached gingiva. Other findings related to this disorder described in the literature are nail changes (peri- and subungeal keratosis), hyperhidrosis of the affected skin and follicular hyperkeratosis (1,4).

On our case, the patient showed non-painful skin lesions only on the soles of her feet and hyperkeratosis on the oral mucosa, no other visible alterations were noticed on the palms of her hands or nails. Transmission of the clinical signs of this disease through at least 3 consecutive generations (grandmother, father and daughter) suggests an autosomal dominant inheritance. The histopathological findings, specially the hyperkeratinization, the basophilic keratohyalin granules and the eosinophilic paranuclear bodies are a hallmark of keratin mutation diseases (7). Based on these features and after excluding other genodermatoses, the final diagnosis was rendered as FPGK.

Although, when Gorlin first described two families affected by FPGK, he suggested a male-to-male transmission of the syndrome, other case reports seem to disagree on this observation (4), and our case is no different. It is important to discard other skin related syndromes that show similar alterations, such as PLS, but because PLS is an autosomal recessive disorder with an early onset in life, usually in the first months after birth resulting in premature tooth loss, it can be clinically differentiated from FPGK (8). Another important differential diagnosis to take into consideration is HES, and autosomal dominant disorder with a 100% penetrance in most cases. Patients with this illness start showing signs of the disease around the second decade of life, developing hyperkeratosis on pressure points on palms and soles (9). Most importantly, patients with HES have about 65% chance of developing esophageal carcinoma by age 60, therefor they need to be placed under regular follow-up (10). Because of the absence of esophageal lesions in our patient and the lack of a familial history of esophageal carcinoma, HES was excluded.

As of the rarity of this syndrome, and because only a few cases have been reported, the clinical features of FPGK and possible accompaniment management remain to be defined in more detail (4), therefore, we encourage clinicians and specialist to report similar cases.
